# Suppression of tomato wilt by cell-free supernatants of *Acinetobacter baumannii* isolates from wild cacao from the Colombian Amazon

**DOI:** 10.1007/s11274-023-03719-9

**Published:** 2023-09-02

**Authors:** Carolina Pisco-Ortiz, Adriana González-Almario, Liz Uribe-Gutiérrez, Mauricio Soto-Suárez, Carol V. Amaya-Gómez

**Affiliations:** 1 Centro de Investigación La Libertad, Corporación Colombiana de Investigación Agropecuaria – Agrosavia, Villavicencio, Meta Colombia; 2 Facultad de Ciencias Agrarias, Universidad Nacional de Colombia, Bogotá, Colombia; 3 Centro de investigación Tibaitatá, Corporación Colombiana de Investigación Agropecuaria-Agrosavia, Mosquera, Cundinamarca Colombia

**Keywords:** Iron-chelators, Bacteria, Antagonistic activity, Biological control, *Fusarium oxysporum* f. sp. *lycopersici*, Plant response elicitation

## Abstract

**Abstract:**

Tomato vascular wilt caused by *Fusarium oxysporum* f. sp. *lycopersici* (*Fol*) is one of the most limiting diseases of this crop. The use of fungicides and varieties resistant to the pathogen has not provided adequate control of the disease. In this study, siderophore-producing bacteria isolated from wild cocoa trees from the Colombian Amazon were characterized to identify prominent strategies for plant protection. The isolates were taxonomically classified into five different genera. Eight of the fourteen were identified as bacteria of the *Acinetobacter baumannii* complex. Isolates *CBIO*024, *CBIO*086, *CBIO*117, *CBIO*123, and *CBIO*159 belonging to this complex showed the highest efficiency in siderophore synthesis, producing these molecules in a range of 91–129 µmol/L deferoxamine mesylate equivalents. A reduction in disease severity of up to 45% was obtained when plants were pretreated with *CBIO*117 siderophore-rich cell-free supernatant (SodSid). Regarding the mechanism of action that caused antagonistic activity against *Fol*, it was found that plants infected only with *Fol* and plants pretreated with SodSid *CBIO*117 and infected with *Fol* showed higher levels of *PR1* and *ERF1* gene expression than control plants. In contrast, *MYC2* gene expression was not induced by the SodSid *CBIO*117 application. However, it was upregulated in plants infected with *Fol* and plants pretreated with SodSid *CBIO*117 and infected with the pathogen. In addition to the disease suppression exerted by SodSid *CBIO*117, the results suggest that the mechanism underlying this effect is related to an induction of systemic defense through the salicylic acid, ethylene, and priming defense via the jasmonic acid pathway.

**Graphical abstract:**

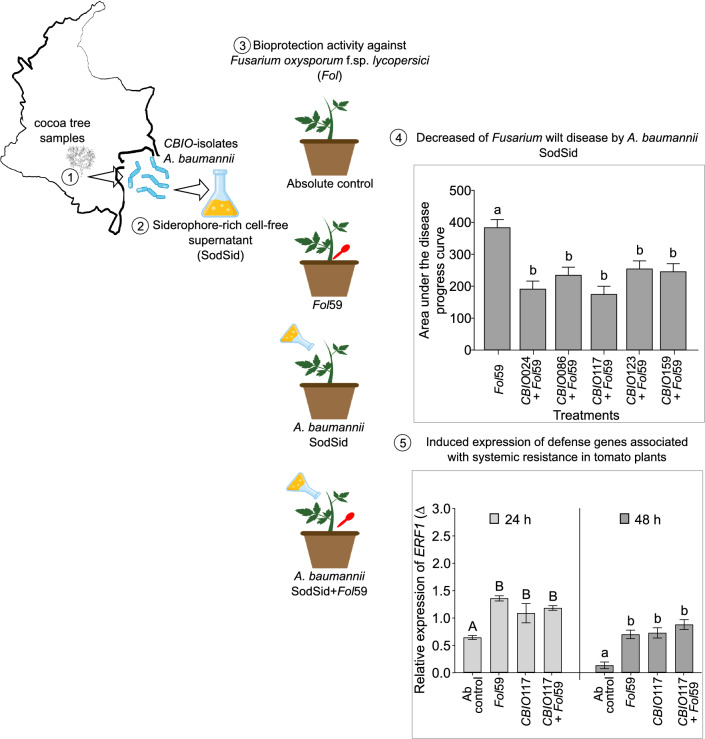

**Supplementary Information:**

The online version contains supplementary material available at 10.1007/s11274-023-03719-9.

## Introduction

Tomato vascular wilt, caused by *Fusarium oxysporum* f. sp. *lycopersici* (*Fol*), is one of the most destructive diseases, causing qualitative and quantitative losses (Atif et al. 2021). Currently, several management strategies are available to control *Fol*, including the use of fungicides and plant-resistant varieties (Amini and Dzhalilov 2010). However, chemical treatment is only available as a protective barrier for seeds (Bodah 2017), and the treatment with agrochemicals to control *Fol* is avoided due to their persistence in soil and their toxicity to beneficial organisms (Yu et al. 2017). Consequently, recent research on its management has focused on alternative strategies such as disease-suppressive soils, beneficial microorganisms, and induction of host resistance (McGovern 2015; Mwangi et al. 2019).

The interest in bacteria for biological control has increased during the last years. These microorganisms exhibit biocontrol of plant diseases, inhibiting the growth of a wide range of plant pathogenic fungi, through the production of siderophores, antimicrobial compounds, and lytic enzymes, and by inducing plant defense responses (Moreno et al. 2018; Karthika et al. 2020). Following exposure to pathogens or beneficial microorganisms, plants activate defense responses both locally and systemically (Mauch-Mani et al. 2017). These responses are associated with Systemic Acquired Resistance (SAR) and Induced Systemic Resistance (ISR). ISR by beneficial microbes is commonly based on priming (Mauch-Mani et al. 2017). Priming is defined as a state of enhanced protection characterized by faster and stronger defense responses against environmental stresses (Martínez-Medina et al. 2016). During beneficial plant–microbe interaction, priming is expressed at the transcriptional level, for instance, elevated levels of expression of *AP2/ERF* family transcription factors have been observed. Several members of this family belong to the signaling cascade that is part of the Jasmonic Acid (JA) and Ethylene (ET)-mediated defense responses (Van der Ent et al. 2009).

Siderophores are iron-chelating agents produced by bacteria, fungi, and plants (Soares 2022). These molecules play a role in the solubilization of iron from minerals that are found as part of insoluble complexes. The excretion of these molecules allows bacteria to sequester the available iron and transport it into the cell. In the environment, the ferric form of iron is insoluble and physiologically unavailable. Microorganisms have evolved to synthesize siderophores that have a high affinity for ferric iron. These ferric iron-siderophore complexes are transported to the cytosol and then reduced to ferrous iron to be accessible to microorganisms (Saha et al. 2016). Siderophore production in *Acinetobacter* and other bacteria genera is regulated by the concentration of available iron in the growth medium. Maindad et al. (2014) showed that the synthesis of an acinetobactin-like siderophore produced by *Acinetobacter calcoaceticus* strain HIRFA32 decreases as iron concentration increases. Similarly, the catechol activity of *Acinetobacter baumannii* siderophores was found to reach 70% at a concentration of 20 µM of FeCl_3_ and decreased to 45% at 80 µM of FeCl_3_ (Modarresi et al. 2015). In *Bacillus anthracis,* while production of the siderophore bacillibactin was found to be highly regulated by the concentration of available iron, petrobactin synthesis is likely regulated by additional virulence-related factors (Lee et al. 2011). Dumas et al. (2013) described that *Pseudomonas aeruginosa* switches from the pyochelin production under highly iron-limiting conditions, pyoverdine production when a moderate iron concentration is available.

Besides siderophore's role as iron-bearers, these molecules have been reported to have an antifungal effect or to induce the activation of the plant defense responses, including the activation of ISR (Dellagi et al. 2009; Santoyo et al. 2010; Aznar and Dellagi 2015). Several studies have shown that different siderophore-producing *Pseudomonas* species, under low iron conditions, are associated with plant protection against pathogens, including, *Botrytis cinerea*, *Colletotrichum lindemuthianum*, *Pythium splendens*, *Pseudomonas syringae*, *Magnaporthe oryzae* and *Fusarium oxysporum* (Buysens et al. 1996; Meziane et al. 2005; De Boer et al. 2007; De Vleesschauwer et al. 2008; Aznar et al. 2014). In addition, results obtained by Yu et al. (2011) showed that treatment with bacillibactin, a catechol-like siderophore produced by *Bacillus subtilis* CAS15, resulted in a 50% disease reduction in pepper during infection with *Fusarium oxysporum* f. sp. *capsica*. These studies demonstrated that siderophores can (i) trigger ISR-mediated resistance and/or (ii) suppress the pathogen by uptake of iron from the environment. Recent reports indicate that siderophore-triggered ISR involves the accumulation of reactive oxygen species and phenolic compounds, and the activation of hormone signaling pathways particularly those mediated by ET and JA.

In view of the few studies that have conducted to explore the biological diversity and attributes of microorganisms adapted to megadiverse areas such as the Amazon rainforest (Guerra et al. 2020), this study aimed to characterize siderophore-producing bacteria isolated from this region and to identify promising candidates for the suppression of tomato vascular wilt caused by *Fol*. The expression level of gene markers from the JA, ET, and salicylic acid signaling pathways was measured to better understand the mechanism of antagonistic action exerted by siderophore-rich cell-free supernatant applied to plants.

## Materials and methods

### Bacteria isolates and physiological characterization

The fourteen bacterial isolates used in this study (*CBIO*021, *CBIO*024, *CBIO*086, *CBIO*101, *CBIO*117, *CBIO*118, *CBIO*120, *CBIO*121, *CBIO*123, *CBIO*127, *CBIO*133, *CBIO*142, *CBIO*149, and *CBIO*159) were previously isolated from rhizosphere and phyllosphere samples collected from wild cocoa trees (*Theobroma subincanum* Mart.*, Theobroma cacao* L. and *Herrania nitida* (Poepp.) R.E. Shult.) from the Colombian Amazon. The isolates were deposited in the microbial germplasm bank of Agrosavia. The sampling coordinates are shown in Table S1. Isolates were characterized by Gram staining (Gram 1884). Lactose fermentation was visualized on MacConkey agar (Oxoid, MacConkey, 1985) where individual colonies were plated and incubated at 37 °C for 48 h in the dark. Hemolytic activity was determined qualitatively using blood agar culture medium (Oxoid) containing 5% v/v bovine blood (Lányi, 1987). After 24 h of incubation at 37 °C, the presence around the inoculum site of green or translucent halos, or the absence of halo was interpreted as alpha-hemolysis, beta-hemolysis or gamma-hemolysis, respectively. Experiments were performed in triplicate.

### Quantification of siderophore production

Siderophore production was evaluated in iron-free modified minimal medium (MM) (Robertsen et al. 1981) containing: C_5_H_8_NNaO_4_, 2.42 g/L; C_6_H_12_O_6_, 10 g/L; supplemented with stock solution 10 mL/L of K_2_HPO_4_, 3 mg/mL; KH_2_PO_4_, mg/mL; CaCl_2_·6H_2_O, 0.5 mg/mL; NaCl, 0.5 mg/mL; MgSO_4_, 1,5 mg/mL; pH 7.0). The cell mass of a 24-h culture of each isolate was used to inoculate MM broth to an initial optical density (OD) of 0.2 at 600 nm. The cell culture was incubated at 28 °C under constant shaking at 117 rpm for 72 h with an oscillation diameter of 25 mm (Tecnal®, incubator shaker TE-421, Brazil). The cell-free supernatant was collected by centrifugation at 8273×*g* for 15 min at 4 °C (Eppendorf®, centrifuge 5430, Germany). Detection of siderophores was estimated by Chrome Azurol Sulfonate (CAS) colorimetric test, as described by Schwyn and Neilands, (1987). Briefly, 500 μL of the culture supernatant were mixed with 500 μL of CAS solution (As). After incubation for 1 h in the dark at room temperature, the OD was measured at 630 nm. MM broth without inoculum mixed with CAS solution was used as a control (Ar). Siderophore production was expressed as a color change of the reaction from blue to orange and was calculated by the following equation: $[(\mathrm{Ar }-\mathrm{ As}) /\mathrm{ Ar}] *100$. Where Ar represents the absorbance of the control and As represents the absorbance of the sample. To estimate the siderophore concentration, a standard calibration curve was performed using deferoxamine mesylate (DFOM) as an iron chelator following the methodology described by Radzki et al. (2013) and Mehnert et al. (2017). The concentration was expressed in µmol/L DFOM equivalents. According to Radzki et al. (2013) 1 mmol of DFOM captures 56 mg of Fe^3+^.

### Taxonomic identification and phylogeny

The total deoxyribonucleic acid (DNA) from the fourteen selected isolates was extracted from grown cultures using the MoBio PowerLyzer® UltraClean® Microbial Kit (Carslbad, CA) following the manufacturer's instructions. The 16S V4 of the ribosomal ribonucleic acid (rRNA) gene was amplified by polymerase chain reaction (PCR). The PCR reaction mixture consisted of 5 µL of DNA template, 1 µL (10 µM) of the primers 515F (5ʹ-GTGYCAGCMGCCGCGGTAA-3ʹ) and 806R (5ʹ-GGACTACNVGGGTWTCTAAT-3ʹ) (Apprill et al. 2015; Parada et al. 2016), 10 µL of Go Taq® Green Master Mix (Promega) and 8 µL of nuclease-free water (Invitrogen™). Amplification was carried out by an initial denaturation step at 94 °C for 3 min, followed by 35 cycles of 45 s of denaturation at 94 °C, 60 s of annealing at 50 °C and 90 s of extension at 72 °C (Applied Biosystems SimpliAmp, Thermal Cycler, USA). Final extension was carried out at 72 °C for 10 min. The resulting amplification products were separated using a 1% w/v agarose gel. Sequencing of the amplified DNA fragments was performed by Corpogen Corporation (https://www.corpogen.org/, Bogotá. Colombia). To resolve ambiguous bases on the forward and reverse trace files, the resulting sequences were manually checked considering the PHRED scores received. Taxonomic identification of isolates to genus level was carried out using the NCBI GenBank database (https://blast.ncbi.nlm.nih.gov) and the basic local alignment search analysis tool (BLASTn). The resultant unrooted tree topology was evaluated by bootstrap analyses (Felsenstein 1985) of the neighbor-joining method based on 1000 bootstrap replicates. Trees were rooted using the partial sequence of the 16S rRNA gene of *Lactococcus plantarum* (NR_044358.1) as an outgroup.

### Identification of *Acinetobacter* species by Vitek 2 system compact

The eight isolates identified as *Acinetobacter* sp. were cultured in Luria–Bertani media (LB) for 24 h at 35 °C. A sterile swab was used to transfer and suspend the cell mass in 3.0 mL of sterile saline solution (0.45% NaCl, pH 7.0) in a test tube. The turbidity of the cell suspension was measured and adjusted to the McFarland turbidity range between 0.50 and 0.63 using a DensiChekTM Plus (VITEK® 2, BioMereux, USA). The identification GN ID cards (Ref 21341, VITEK® 2 GN) were inoculated with each microorganism's cell suspension using a Vitek 2 Compact® (BioMerieux, Lyon, France). All cards were incubated at 35 °C for 18 h. Each test reaction was read every 15 min to measure turbidity and colored products of substrate metabolism. Interpretation of the results was carried out as described by Pincus (2006) where the GN card is based on established biochemical methods for the identification of isolates gram-negative bacilli.

### Induction of siderophores production

The production of siderophores in five *A. baumannii* isolates that showed increased ability to excrete these molecules (*CBIO*024, *CBIO*086, *CBIO*117, *CBIO*123, and *CBIO*159) was evaluated in MM broth without iron, and in MM containing a low (22 µM) and a high (220 µM) concentration of FeCl_3_·6H_2_O. A bacterial inoculum was prepared by culturing the selected bacteria on nutrient agar for 24 h at 28 °C. The cell mass was then washed twice with NaCl (0.85% w/v) by centrifugation for 5 min at 4430×*g* (Hermle, centrifuge Z 326 k, Germany), removing the supernatant between washes. The resulting cell pellet was resuspended in MM broth supplemented with each of the FeCl_3_·6H_2_O concentrations and adjusted to a final OD of 2.0 at 600 nm (Thermo Fisher Scientific, Genesys 150 spectrophotometer, USA). Next, a 0.5 mL aliquot of each bacterial inoculum was added to Falcon tubes (15 mL) containing 4.5 mL of the MM broth without iron or supplemented with FeCl_3_·6H_2_O, until a final OD of 0.2 at 600 nm was reached. Cell cultures were incubated at 28 °C under constant shaking at 180 rpm with an oscillation diameter of 10 mm for 72 h (Heidolph, Shakers Unimax 1010, Germany). Siderophore production was quantified using CAS assay as described above. Three biological replicates were performed in a series of 4 samples.

### Bioactivity assays against *Fol*

The bioactivity of the cell-free supernatant of the five *A. baumannii* isolates *CBIO*024, *CBIO*086, *CBIO*117, *CBIO*123, and *CBIO*159 was evaluated against *Fol* by in vivo experiments on tomato plants. For this purpose, the pathogen *Fusarium oxysporum* f. sp. *lycopersici* Race 2 identified as *Fol*59 was used in this study (Carmona et al. 2020; Yu et al. 2022).

Cell-free supernatant of each isolate with high content of siderophores (SodSid) was produced in MM without iron using the methodology described above. For this experiment, twenty-eight days old seedlings of the susceptible tomato cultivar Santa Cruz Kada grown in a growth chamber at a temperature of 28 °C, with a relative humidity of 60% and a photoperiod of 14 h of light and 10 h of darkness were used. Plants were treated by drenching with 10 mL of SodSid of each isolate. Forty-eight hours after SodSid treatment, plants were removed from the seedbed and infected with *Fol*59 by root immersion into a suspension of 1 × 10^5^ microconidia/mL (Carmona et al. 2020). Subsequently, the plants were transplanted into 16 oz pots containing soil and sand in a 2:1 ratio. Treatments consisted of plants inoculated only with SodSid of *A. baumannii* isolates (*CBIO*024, *CBIO*086, *CBIO*117, *CBIO*123, and *CBIO*159) and plants inoculated with SodSid and infected with *Fol*59 (*CBIO*024 + *Fol*59, *CBIO*086 + *Fol*59, *CBIO*117 + *Fol*59, *CBIO*123 + *Fol*59, and *CBIO*159 + *Fol*59). Water-treated plants were considered as absolute control (Ab control), and plants infected with *Fol*59 (*Fol*59) were used as pathogen control. The experimental setup was a randomized complete factorial design with two factors. The first factor corresponded to the five bacterial supernatants, the second to plants with or without the pathogen infection, for a total of 10 treatments and two controls. Three biological replicates were performed. Each biological replicate comprised three technical replicates, and each technical replicate consisted of eleven plants. Selected parameters related to pathogenicity, plant growth, and regulation of ISR-associated genes were evaluated.

Disease incidence and severity were recorded periodically for 21 days. To assess disease severity, the scale proposed by Rongai et al. (2017) was used and modified as follows: absence of symptoms was recorded as level 0; slight yellowing appearance of one or two leaves was recorded as level 1; necrotic lesions formed both, at the base of the stem and on internodes in the center of the plant, with or without the yellowing appearance of basal and median leaves were recorded as level 2; total yellowing appearance of basal leaves, with some wilted leaves and necrotic lesions in the steam extending upwards was recorded as level 3; loss of turgor and wilting of the plant was recorded as level 4; a dead plant was recorded as level 5. The severity index (SI) and area under the disease progression curve (AUDPC) were calculated using the equations described by Chiang et al. (2017) and Pedroza-Sandoval and Gaxiola (2009). The plant height, dry weight, and chlorophyll content of plants were determined 16 days post-inoculation with *Fol*59 (dpi). The plant height was recorded using a tape measure. The weight of six plants per treatment was measured after drying in an oven at 60 °C for 72 h. Chlorophyll content was estimated in Soil Plant Analysis Development (SPAD) units using the Minolta® SPAD 502 chlorophyll meter. Leaf chlorophyll content was measured in the distal part of the adaxial side of an apical leaf (three measurements) (Hurtado et al. 2017), values shown are means ± SE (n = 11) plants, using the mean of three measurements per plant.

### Quantification of the transcription of systemic resistance-related genes

The relative expression of three defense marker genes was calculated by quantitative Real-Time Reverse Transcription Polymerase Chain Reaction (qRT-PCR). The treatments evaluated were (i) non-inoculated plants treated with water (Ab control), (ii) plants only inoculated with *CBIO*117 SodSid (*CBIO*117), (iii) plants only infected with *Fol*59 (*Fol*59, pathogen control), and iv) plants inoculated with *CBIO*117 SodSid and infected with *Fol*59 (*CBIO*117 + *Fol*59). The differential expression of *PR1*, *ERF1*, and *MYC2* genes associated with SA, ET, and Jasmonic acid (JA), respectively, was assessed at 24 and 48 h post infection with *Fol* (hpi). For this, specific primers previously reported for *PR1, ERF1,* and *MYC2* were used, additionally, elongation factor expression (EF1a) was used as a reference (Table 1) (Martínez-Medina et al. 2013).

**Table 1:** Primer sequences for defense marker genes in qPCR analysis

Primer	Sequence (5ʹ–3ʹ)	References
LePR1aqF LePR1aqR	GTGGGATCGGATTGATATCCT CCTAAGCCACGATACCATGAA	Martínez-Medina et al. (2013)
LeERF1qF LeERF1qR	GAGGGGTCCTTGGTCTCTACTC ACAGCAGCTGGAGATAATCCAT	Huang et al. (2004)
LeMYC2qF LeMYC2qR	AGCAGGAGCATCGGAAGAA CCAAATCGGGCTGGAACTA	Martínez-Medina et al. (2013)
LeEF1aqF LeEF1aqR	GATTGGTGGTATTGGAACTGTC AGCTTCGTGGTGCATCTC	Martínez-Medina et al. (2013)

Total RNA extraction was performed following the protocol described by Yockteng et al. (2013). Complementary DNA (cDNA) synthesis was performed using the ProtoScript® II First Strand cDNA kit (New England BioLabs) following the manufacturer's recommended protocol. Quantitative RT-PCR reactions were performed using the IqTM SYBR® Green Supermix kit (Bio-Rad®) in a final volume of 10 μL per reaction. The reaction mixture consisted of 2 μL of cDNA, 0.4 µL of each primer (0.4 μM), 5 µL of 2X of IQ SYBR Green Supermix, and 2.2 μL of molecular grade water. Reactions were performed in duplicate for each of the three independent biological replicates. To analyze the relative gene expression data, the common-base method proposed by Ganger et al. (2017) was used according to the following equation: $\Delta \mathrm{Ct}=({\left(\mathrm{Log}\right)}_{10}{\mathrm{E}}_{\mathrm{ref}}\times{\mathrm{Ct}}_{\mathrm{ref}} )-({\left(\mathrm{Log}\right)}_{10} {\mathrm{E}}_{\mathrm{Goi}}\times{\mathrm{Ct}}_{\mathrm{Goi}} )$ where E_ref_ is the efficiency of the housekeeping gene *EF1*a, E_Goi_ is the efficiency of the target gene, and Ct are threshold values of the cycle.

### Antimicrobial activity test in dual-liquid culture assay

To test antimicrobial activity by the dual-liquid culture method, 50 mL of iron-deficient media containing glucose at 5% w/v (IDM, Muller et al. 1984) was taken in a 250-mL culture flask and inoculated with 50 mL of *CBIO*117 inoculum (10^7^ cells/mL) and a 5 mm size PDA plug of freshly grown *Fol*59. The culture flasks were incubated at 28 °C for 72 h on a rotary shaker at 120 rpm with an oscillation diameter of 19 mm (Max^Q^ 4000, Barnstead/Lab-line, Switzerland). *Fol*59 cultures without *CBIO*117 inoculum were used as controls. After incubation, cell culture supernatants were filtered through a Whatman No. 1 filter paper (1001-055, Whatman) and dried at 60 °C. *Fol*59 biomass growth on both cultures, co-inoculated with *CBIO*117 and control (without *CBIO*117), was compared to assess the antagonistic activity of *CBIO*117. The percentage reduction in fungal biomass was calculated by the equation: $Inhibition \left(\%\right)=\frac{\left({W}_{1 }{-W}_{2}\right)}{{w}_{1} \times 100}$ where W_1_ corresponds to the biomass (g) of *Fol*59 in the control culture, while W_2_ corresponds to the biomass of *Fol*59 grown in co-culture with *CBIO*117 (Basha and Ulaganathan 2002; Trivedi et al. 2008).

### Statistical analysis

To evaluate the effect of treatments on disease severity, a linear mixed-effects model analysis was performed. Treatment, sampling day, biological replication, and interaction were considered fixed effects. The experimental unit (plant) was included as a random effect (Schandry 2017). Relative chlorophyll content was analyzed using linear mixed-effects models. Plant height and dry weight values were analyzed by ANOVA and Tukey test. Tukey tests for pairwise comparisons of means were performed at a significance level of *P* < 0.05. Relative expression levels were analyzed using cycle threshold values calculated by univariate analysis at each post-inoculation time point (Popović et al. 2021). All statistical analyses were carried out using R software (version 4.1.2).

## Results

### Phenotypic characterization of bacterial isolates

The fourteen isolates used in this study were previously selected for their ability to produce siderophores. For each isolate, geographic location, plant part for sample collection (rhizosphere or phyllosphere), and phenotypic characteristics are shown in Table 2. The isolates showed differential microscopic characteristics and ability to ferment lactose and lyse blood cells. Two isolates were Gram-positive bacilli, eight were Gram-negative coccobacilli, and four were Gram-negative bacilli. The lactose utilization assay showed that most isolates fermented lactose as observed by the appearance of a clear halo around colonies. The greatest diversity was found in the hemolytic activity test, in which isolates showing α, β, and γ activity were identified.

**Table 2:** Geographical location, plant part of sample collection (rhizosphere or phyllosphere) and phenotypic characteristics of isolates analyzed in this study

Isolate code	Isolation origin			Phenotypic characteristics			
	Location	Plant part	Plant Host	Gram stain	Morphology	Lactose	Hemolysis
*CBIO*021	Caquetá	Rh	*T. subincanum*	G+	Bacilli	Ng	β
*CBIO*024	Caquetá	Rh	*T. subincanum*	G−	Cocco	–	γ
*CBIO*086	Caquetá	Phl	*T. subincanum*	G−	Cocco	+	γ
*CBIO*101	Caquetá	Rh	*T. subincanum*	G−	Bacilli	–	α
*CBIO*117	Putumayo	Rh	*T. subincanum*	G−	Cocco	–	γ
*CBIO*118	Caquetá	Rh	*T. subincanum*	G−	Bacilli	–	γ
*CBIO*120	Putumayo	Phl	*T subincanum*	G−	Bacilli	–	γ
*CBIO*121	Putumayo	Phl	*H. nitida*	G+	Bacilli	Ng	β
*CBIO*123	Putumayo	Rh	*T. subincanum*	G−	Cocco	+	γ
*CBIO*127	Putumayo	Phl	*T. subincanum*	G−	Cocco	+	γ
*CBIO*133	Putumayo	Phl	*T. cacao*	G−	Cocco	+	α
*CBIO*142	Putumayo	Phl	*T. cacao*	G−	Bacilli	+	γ
*CBIO*149	Putumayo	Phl	*T. cacao*	G−	Cocco	+	β
*CBIO*159	Caquetá	Phl	*T. cacao*	G−	Cocco	+	γ

*Rh* rhizosphere, *Phl* phyllosphere, *Coccobacilli* Cocco, *G*+ Gram positive, *G-* Gram negative, *Ng* absence of growth

### Quantification of siderophore production

**Fig. 1:**
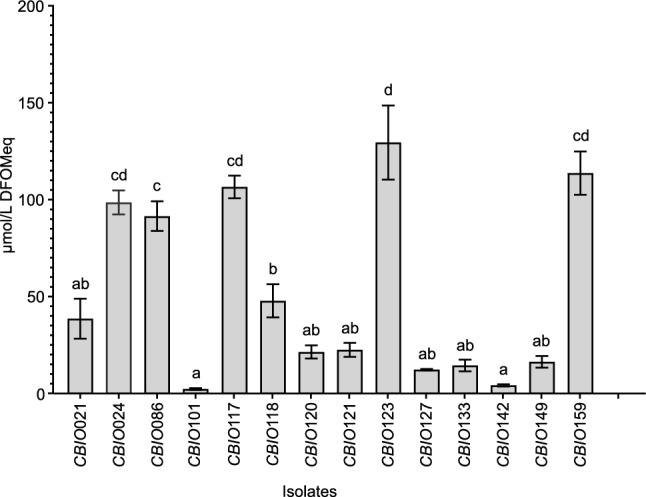
Production of siderophores by *CBIO-*isolates (expressed in µmol/L equivalents of DFOM) after 72 h after incubation. Bacteria were grown in minimal media without iron. Values followed by the same letter do not differ significantly (Tukey test, *P* < 0.05). Values correspond to the mean (± ES) of three biological replicates with n = 3

**Fig. 2:**
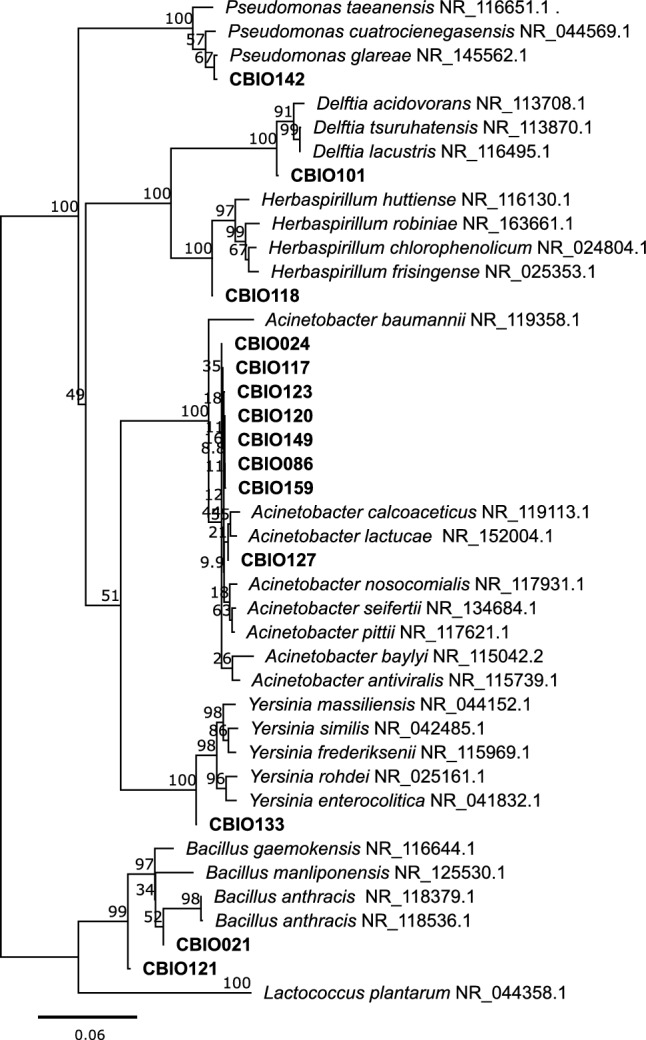
Phylogenetic-tree construction using the neighbor-joining method. The phylogenetic analysis was performed with partial 16S rRNA gene sequences. Bootstrap values, expressed as percentages of 1000 replications, are shown at branch points. The numbers above the branches indicate the percentage of consensus support. Bold letters highlight the *CBIO*-isolates characterized in this study. The tree was rooted using *Lactococcus plantarum* NR_044358.1 as an outgroup

Comparison of siderophore production among 14 siderophore-producing isolates showed significant differences (*P* < 0.05, Fig. 1). The concentration of siderophores ranged from 2.7 µM ± 0.3 (*CBIO*101) to 129.9 µM ± 19.1 (*CBIO*123). Isolates *CBIO*024, *CBIO*086, *CBIO*123, *CBIO*117, and *CBIO*159 showed the highest siderophore production. For these isolates, siderophore concentrations were 99 ± 6.2, 91 ± 7.6, 129 ± 19.1, 107 ± 5.8, and 114 ± 11.1 µmol/L DFOM equivalents, respectively (Fig. 1). For isolates identified as *Delftia* and *Pseudomonas* (*CBIO*101 and *CBIO*142), low concentrations of siderophore (4.6 ± 0.5 µmol/L DFOM equivalents) were observed (Fig. 1 and Fig. 2).

### Taxonomic characterization

Comparison of the partial 16S rRNA gene sequence and phylogeny analysis allowed taxonomic identification of the isolates to genus level. These analyses classified the isolates into 14 genera, eight belonging to the *Acinetobacter* genus, two to the *Bacillus* genus, one to the *Delftia* genus, one to the genus *Herbaspirillum*, one to the genus *Pseudomonas*, and one to the genus *Serratia* (Fig. 2).

**Fig. 3:**
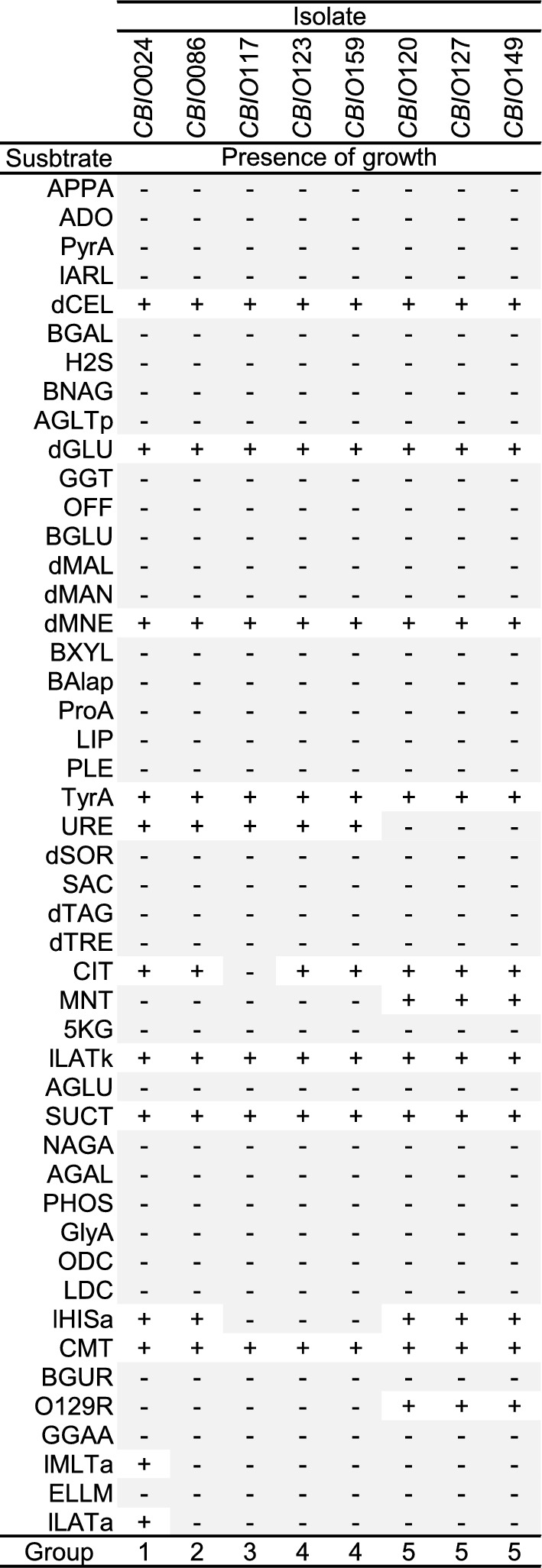
Metabolic profile using ID-GNB card of the VITEK 2 system for taxonomic identification of the *Acinetobacter* sp. isolates. Presence of growth (+), absence of growth (−). Ala-phe-pro-arylamidase (APPA), Adonitol (ADO), l-pyrrolydonyl-arylamidase (PyrA), l-arabitol (IARL), d-cellobiose (dCEL), Beta-galactosidase (BGAL), H2S production (H2S), Beta-N-acetyl-glucosaminidase (BNAG), Glutamyl arylamidase pNA (AGLTp), d-glucosa (dGLU), Gamma-glutamyl transferase (GGT), Fermentation of glucose (OFF), Beta-glucosidase (BGLU), d-maltose (dMAL), d-mannitol (dMAN), d-mannose (dMNE), Beta-xylosidase (BXYL), Beta-alanine arylamidase pNA (BAIap), l-proline-arylamidase (ProA), Lipase (LIP), Palatinose (PLE), Tyrosine arylamidase (TyrA), Urease (URE), d-sorbitol (dSOR), Saccharose/sucrose (SAC), d-tagatose (dTAG), d-trehalose (dTRE), Citrate (Sodium) (CIT), Malonate (MNT), 5-Keto-D-gluconate (5 KG), l-lactate alkalinization (ILATk), Alpha-glucosidase (AGLU), Succinate alkalinization (SUCT), Beta-N-acetyl galactosaminidase (NAGA), Alpha-galactosidase (AGAL), Phosphatase (PHOS), Glycine arylamidase (GlyA), Ornithine descarboxylase (ODC), Lysine decarboxylase (LDC), l-histidine assimilation (IHISa), Cumarate (CMT), Beta-glucuronidase (BGUR), O/129 Resistance (comp.vibrio.) (O129R), Glu-Gly-Arg-arylamidase (GGAA), l-malate assimilation (IMLTa), Ellman (ELLM), l-lactate assimilation (ILATa)

Vitek tests were performed to further characterize the taxonomic identity of the *CBIO* isolates classified within the genus *Acinetobacter*. Isolates *CBIO*024, *CBIO*086, *CBIO*117, *CBIO*123, *CBIO*159, *CBIO*120, *CBIO*127, and *CBIO*149 were classified within the *A. baumannii* complex with a 99% of probability. All these isolates showed five different metabolic patterns (Group 1, 2, 3, 4, and 5; Fig. 3). Most of them were able to grow using d-cellobiose, d-glucose, d-mannose, tyrosine arylamidase, l-lactate, succinate, and coumarate as carbon source. *CBIO*117 was the only isolate unable to grow using citrate as the sole carbon source, and inorganic ammonium salt as the sole source of nitrogen. Remarkably, *CBIO*117, *CBIO*123, and *CBIO*159 did not assimilate l-histidine and were the isolates with the highest siderophore production. In contrast, isolates *CBIO*120, *CBIO*127, and *CBIO*149 showed lower production of iron chelators and did not grow in the presence of urease (Fig. 3).

*A. baumannii* isolates *CBIO*024, *CBIO*086, *CBIO*117, *CBIO*123, and *CBIO*159 were selected for further studies, based on their increased ability to produce siderophores and differences in metabolic profile compared to other isolates.

### Effects of cell-free supernatants of *A. baumanii* isolates on the suppression of vascular wilt

We further confirmed the effect cell-free supernatants of *A. baumanii* on plant protection against *Fol.* To ensure that bioactivity assays were performed with siderophore-rich supernatants, siderophores production was evaluated in MM containing different concentrations of FeCl_3_·6H_2_O. The results showed that at higher iron concentrations, siderophore production decreased (Figure S1). When *A. baumannii* isolates *CBIO*024, *CBIO*086, *CBIO*117, *CBIO*123, and *CBIO*159 were culture in MM without iron, we found that the mean concentration of siderophores production was 43 µmol/L DFMO equivalents. Thus, activities against tomato *Fusarium* wilt were attempted using the cell-free supernatant of selected *CBIO*-isolates grown under iron-starved conditions (SodSid).

**Fig. 4:**
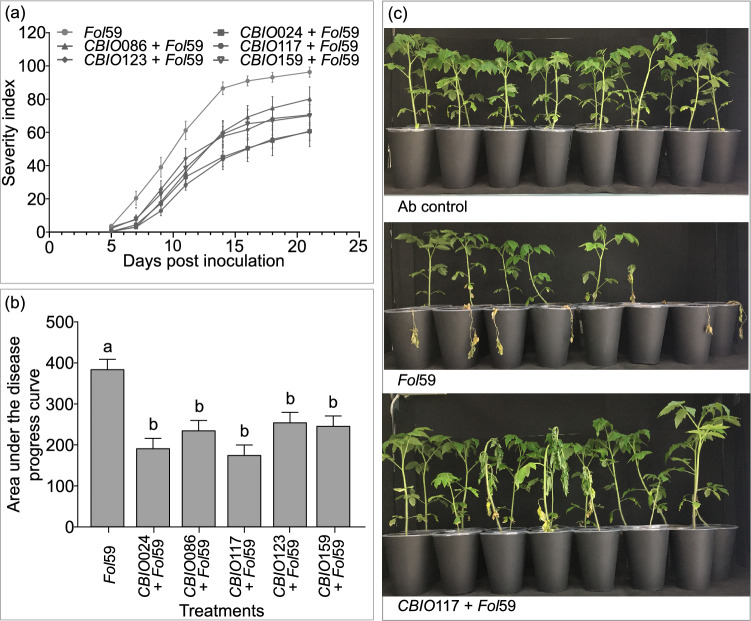
Effect of preventive application of cell-free supernatant from *A. baumanii* isolates on *Fusarium* wilt of tomato. **A** Disease severity index. **B** Effect of SodSid from five *Acinetobacter* sp. isolates on AUDPC severity at 21 days post inoculation. **C** Representative images of tomato plants treated with water (Ab control), infected with *Fol*59 (*Fol*59), and plants previously treated with *CBIO*117 SodSid, subsequently infected with *Fol*59 (*CBIO*117 + *Fol*59). Values correspond to the mean of three biological replicates with n = 15. Value means followed by the same letter do not differ significantly (Tukey test, *P* < 0.05)

Preventive application of SodSid of *A. baumanii* isolates reduced disease incidence in plants infected with *Fol*59 by 31% (*CBIO*024 + *Fol*59), 11% (*CBIO*086 + *Fol*59), 33% (*CBIO*117 + *Fol*59), 24% (*CBIO*123 + *Fol*59) and 20% (*CBIO*159 + *Fol*59) relative to plants only infected with the pathogen, where 100% incidence was reached at 21 dpi. Disease severity index and AUDPC of plants pretreated with SodSid and subsequently infected with *Fol*59 (*CBIO* + *Fol*59) were lower compared to plants infected only with *Fol*59. However, at 21 dpi, the highest disease suppression was observed in *CBIO*117 + *Fol*59 treatment wheredisease severity was reduced up to 45% (Fig. 4B).

### Growth parameters of tomato plants treated with cell-free supernatant of *A. baumannii* isolates

Next, we investigated whether *A. baumannii* SodSid affected plant development rate. The plant height at 16 dpi was significantly delayed (*P* < 0.05) in *Fol*59-infected plants compared to water-only treated plants (Ab control plants), which showed greater increase in height, and higher relative chlorophyll content (Table 3). Interestingly, plants treated only *CBIO*117 SodSid showed the greatest plant height, with significant differences with respect to the other treatments (*P* < 0.05). Plants treated with SodSid of isolate *CBIO*024, SodSid of *CBIO*123 and infected with *Fol*59 (*CBIO*123 + *Fol*59), and plants infected only with *Fol*59 showed lower amounts of total dry matter accumulation. As for the SPAD chlorophyll index, significantly higher SPAD units were observed in all plants treated with SodSid from *CBIO*-isolates and plants treated with SodSid of the *CBIO*-isolates and then infected with *Fol*59, compared to Ab and *Fol*59 control plants (*P* < 0.05).

**Table 3:** Growth parameters of tomato plants treated with cell-free supernatant of *A. baumannii* isolates

Treatments	Plant height* (cm)	Dry weight* (mg)	Chlorophyll* (SPAD)
Ab control	20.6 ± 0.5^Aa^	190 ± 0.02^Aab^	24.7 ± 0.3^Aa^
*CBIO*024	20.2 ± 0.5^Aa^	120 ± 0.02^Aa^	26.2 ± 0.3^Ab^
*CBIO*086	21.8 ± 0.5^Aab^	170 ± 0.02^Aab^	26.2 ± 0.3^Ab^
*CBIO*117	23.5 ± 0.5^Ab^	220 ± 0.02^Ab^	27.1 ± 0.3^Ab^
*CBIO*123	21.6 ± 0.5^Aab^	180 ± 0.02^Aab^	27.1 ± 0.3^Ab^
*CBIO*159	21.3 ± 0.5^Aa^	170 ± 0.02^Aab^	27.0 ± 0.3^Ab^
*Fol*59	19.5 ± 1.0^Ba^	90 ± 0.02^Aa^	22.8 ± 0.3^Ba^
*CBIO*024 + *Fol*59	18.8 ± 0.6^Ba^	170 ± 0.02^Aab^	24.3 ± 0.3^Bb^
*CBIO*086 + *Fol*59	19.7 ± 0.7^Ba^	130 ± 0.02^Aab^	24.3 ± 0.3^Bb^
*CBIO*117 + *Fol*59	19.5 ± 0.6^Ba^	160 ± 0.02^Aab^	25.2 ± 0.3^Bb^
*CBIO*123 + *Fol*59	18.4 ± 0.7^Ba^	90 ± 0.02^Aa^	25.2 ± 0.3^Bb^
*CBIO*159 + *Fol*59	18.3 ± 0.7^Ba^	140 ± 0.02^Aab^	25.1 ± 0.3^Bb^

*Each variable was analyzed independently. Absolute control (Ab control), plants infected with *Fol*59 (*Fol 59*). The mean (+ ES) of plant height and relative chlorophyll content values correspond to three biological replicates with n = 15. The mean (+ ES) of dry weight values correspond to three biological replicates with n = 6. Value means followed by the same letter do not differ significantly (Tukey test, *P* < 0.05). Capital letters show the significant differences between plants not infected and infected with *Fol*59. Lower-case letters show significant differences between the treatments and their respective control

### Effect of SodSid on the expression of defense marker genes in *Fol*-infected tomato plants

**Fig. 5:**
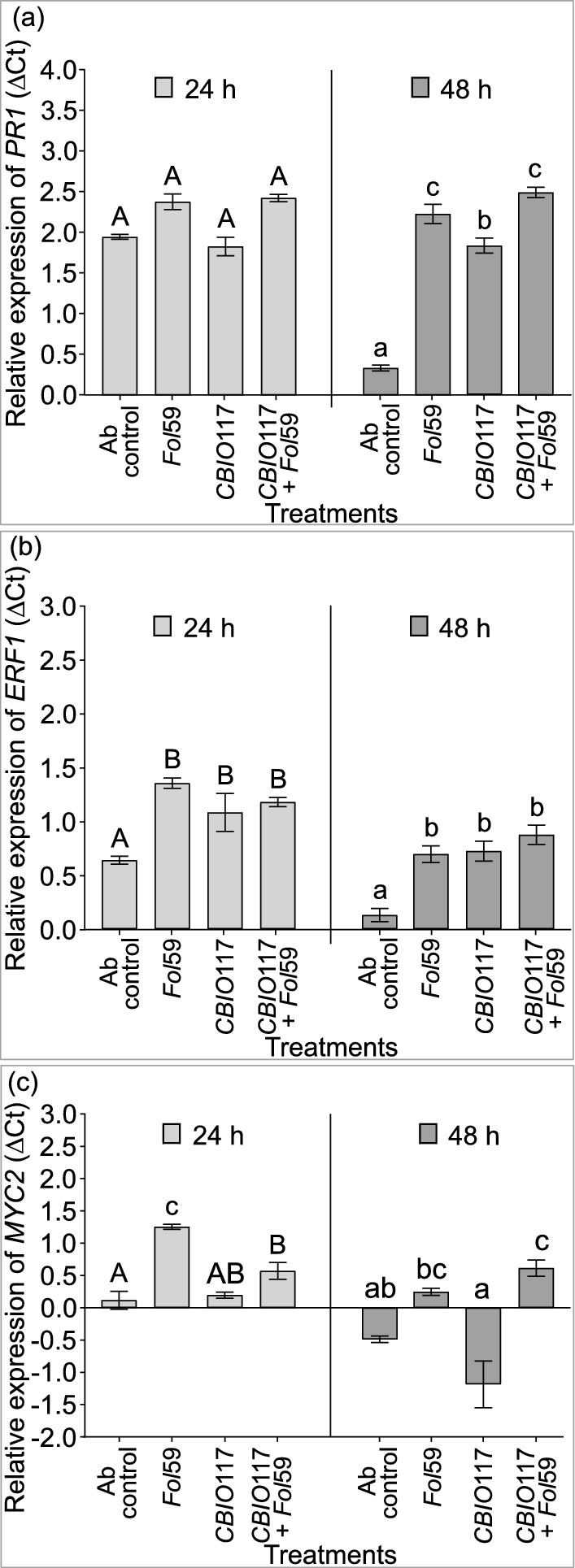
Relative expression of defense genes to assess the effect of cell-free supernatant (SodSid) of *A. baumannii CBIO*117 on the activation of tomato defense responses. **A** Relative expression of *PR1* gene. **B** Relative expression of the *ERF1* gene, and **C** Relative expression of *MYC2* gene. Treatments correspond to plants treated with water (Ab control), plants infected with *Fol*59 (Fol59), plants treated with SodSid of *CBIO*117 isolate (*CBIO*117), and plants treated with SodSid of *CBIO*117 and then infected with *Fol*59 (*CBIO*117 + *Fol*59). Values correspond to the mean (± ES) of two biological replicates with n = 2. Means of values followed by the same letter do not differ significantly (Tukey test, *P* < 0.05)

To determine whether the reduction of wilt disease severity in tomato plants treated with SodSid from *A. baumannii CBIO*117 is related to the induction of plant defense genes, the relative expression of the *PR1, ERF1*, and *MYC2* genes was evaluated in plants treated only with SodSid from *CBIO*117 isolate and plants pretreated with SodSid and subsequently infected with *Fol*59. Two separate time points were considered after infection with *Fol*59 (24- and 48-h post-infection (hpi)). At the earliest time (24 hpi), there was no significant difference in *PR1* expression levels between treatments. However, at 48 hpi all plants, including those treated only with SodSid from *A. baumannii CBIO*117 showed a significant up-regulation of *PR1* gene (*P* < 0.05) (Fig. 5A). These results indicate SodSid application of *A. baumannii CBIO*117 is able to induce plant defense responses, including *PR1*-based basal immunity (Fig. 5A).

Similarly, *ERF1* gene expression levels increased in both conditions, after treatment *CBIO*117 SodSid alone and in plants pretreated with SodSid and subsequently infected with *Fol*59, compared with control plants. This means that the preventive application on tomato roots with SodSid of *CBIO*117 promoted ethylene-mediated defense responses in tomato plants (Fig. 5B).

Expression of the *MYC2* transcription factor was not induced after preventive SodSid application of *A. baumannii CBIO*117 on tomato roots. The highest level of *MYC2* expression occurred at 24 hpi in plants infected only with *Fol*59, followed by plants pretreated with SodSid and then infected with the pathogen (*CBIO*117 + *Fol*59). However, at 48 hpi, a decrease in *MYC2* gene expression was detected in plants infected with *Fol*59, whereas the gene expression was maintained in plants previously stimulated with the SodSid and infected with the pathogen (*CBIO*117 + *Fol*59).

### Antimicrobial activity assay in dual culture in liquid medium

To gain further insight into the antimicrobial activity of *A. baumannii CBIO*117, we performed an assay using the dual culture method in a liquid medium. It was observed that *CBIO*117 isolate was not able to inhibit the growth of *Fol*59. No significant differences (*P* < 0.05) were observed when comparing *Fol*59 biomass growth in either culture, co-inoculated with *CBIO*117 (0.20 ± 0.08 g/mL) or the control culture without *CBIO*117 (0.22 ± 0.06 g/mL).

## Discussion

The tomato crop is threatened by wilt caused by *Fusarium oxysporum* f. sp. *Lycopersici*. Increasing attention is being paid to reducing the use of synthetic fungicides to control plant diseases, mainly because of their negative impact on production costs, environmental pollution, risks to human health, development of fungicide resistance, and post-harvest fruit quality. The use of beneficial microorganisms or their metabolites is a promising alternative for disease management. In this study, siderophore-producing bacteria from the rhizosphere and phyllosphere of wild cocoa trees collected in the Colombian Amazon were characterized. Furthermore, the bioactivity of the cell-free supernatants containing siderophores was tested against *Fol*.

Fourteen isolates showed high siderophore production, of which eight were identified as *A. baumannii*. Several structurally distinct siderophores have been described for this bacterium including acinetobactin, baumannoferrins A and B and, fimsbactins A–F. A large collection of well-characterized siderophores has been also described in the same bacterial genus where the remaining *CBIO-*isolates were classified. For instance, serobactin A, B, and C from *Herbaspirillum* (Rosconi et al. 2013; Tejman-Yarden et al. 2019), bacillibactin and petrobactin from *Bacillus* (Wilson et al. 2006), serratiochelin, chrysobactin, aerobactin, and enterobactin from *Serratia* (Ehlert et al. 1994; Weakland et al. 2020), and pyoverdine, ferrioxamine, piocheln, and ferribactin from *Pseudomonas* (Aguilar et al. 2021). The production of several structurally diverse iron chelators by the same bacteria has been associated with improved iron acquisition and prevention of iron deprivation under different environmental conditions, facilitating the uptake of other metals, and contributing to competitiveness (McRose et al. 2018). Given that the *CBIO*-isolates were isolated in iron-depleted media, it could be considered that the ability to produce structurally diverse siderophores favors the prevalence of the *A. baumannii* isolates, explaining why a higher representative number of isolates from this genus was obtained. To better understand the ability of *CBIO*-isolates to survive under iron-limited conditions, their growth and persistence under iron deficient and iron-abundant conditions need to be evaluated.

Plant protection activity of *Acinetobacter* sp. bacteria against phytopathogenic filamentous fungi and bacteria has been reported before (Xue et al. 2009; Safdarpour and Khodakaramian 2018; Foughalia et al. 2022; Khalil et al. 2021). Different molecules are associated with suppression of these fungi, including siderophores, gibberellic—and indole acetic acids, volatile compounds, or microbial enzymes such as chitinases and proteases. In this study, the application of cell-free supernatant reduced the incidence and severity of the *Fusarium* wilt of tomato. Further studies are needed to better understand the metabolites present in the supernatant of the *CBIO*-isolates that generated the plant protection activity. However, as these supernatants were rich in siderophores, it is possible that the antagonistic activity was at least partially generated by these molecules. Prashant et al. (2009) demonstrated the antifungal activity of catechol-like siderophores from *Acinetobacter calcoaceticus* strain SCW1 against *Aspergillus flavus*, *Aspergillus niger*, *Colletotrichum capsicum*, and *F. oxysporum*. Sayyed and Reddy (2011) reported the growth inhibitory potential of siderophore-rich cell-free supernatants obtained from *Acinetobacter* sp. SH-94B isolate against *A. niger* NCIM 1025, *A. flavus* NCIM 650, *F. oxysporum* NCIM 1281, *Alternaria alternata* ARI 715, *Cercospora arachichola*, *Metarhizium anisopliae* NCIM 1311 and *Ralstonia solanacerum* NCIM 5103. Bacterial siderophores have already shown to play a role in antagonizing plant pathogens through iron deprivation, but also by activating plant-induced systemic resistance (Höfte and Bakker 2007; Betoudji et al. 2020). A synthetic siderophore of fimsbactin-like structure produced by *Acinetobacter* sp. was reported as an iron-bearer under iron deprivation conditions and induce systemic priming in *Arabidopsis thaliana* (Betoudji et al. 2020). Since siderophore-rich cell-free supernatants from *CBIO*-isolates were used, the hypothesis regarding competition for iron as a plant protection mechanism seems unlikely. However, as the cell supernatant was applied before infection with *Fol*59, and gene expression analysis showed differences between plants inoculated with the supernatants alone (SodSid) with respect to absolute control plants, the bioactivity could be better explained by induction of systemic resistance. In addition, the absence of antimicrobial activity was observed when *CBIO*117 supernatant was directly confronted against *Fol,* indicating that disease suppression might not be associated with the action of antimicrobial compounds.

Plants respond to biotic stresses caused by pathogens and pests through a localized defense response at the site of infection or, sometimes, by a systemic response known as SAR. However, some beneficial microorganisms that mainly inhabit the rhizosphere can induce systemic resistance in the plant to counteract phytopathogen attack and strengthen physiological processes (Romera et al. 2019). Secretion of molecules such as siderophores by beneficial microorganisms has been shown to improve plant nutrition and trigger plant defense response (Verbon et al. 2017). In this study, SodSid *CBIO*117 activated SA and ET signaling pathways in tomato plants, as evidenced by changes in gene expression of *PR1* and *ERF1*. These genes did not decrease in expression after infection with the pathogen. However, early expression of *PR1* and *ERF1* in plants only infected with the pathogen could be associated with PAMP-triggered immunity, in which PR1 protein accumulation could potentially restrain the pathogen and induce activation of defense-related pathways (Boccardo et al. 2019). These results suggest that the application of siderophore-rich cell-free supernatants prior to pathogen infection elicits a transient systemic response to counteract plant pathogen attack via the AS and ET signaling pathways. Interestingly, no changes were detected in the expression of the *MYC2* gene marker of the jasmonic acid pathway upon SodSid *CBIO*117 application. However, when plants previously treated with SodSid were infected with *Fol*59, the *MYC2* gene increased expression levels. This response may be associated with an induction of priming defense, reinforcing plant defenses against pathogen attack.

In conclusion, this study demonstrates that siderophore-rich cell-free supernatants of *A. baumannii CBIO*117 can activate systemic resistance in tomato plants and could generate a priming-type defense against *Fol*. This siderophore-producing bacterium could be, in the near future, a potential biocontrol agent to control plant diseases. To elucidate the hormonal signaling pathway involved in the plant protection response, evaluation of additional genes involved in systemic resistance is recommended.

### Supplementary Information

Below is the link to the electronic supplementary material.

Supplementary file1 (DOCX 102 kb)

## Data Availability

The datasets generated during the current study are available from the corresponding author upon reasonable request.
