# Supplementary material for: Suppression of tomato wilt by cell-free supernatants of Acinetobacter baumannii isolates from wild cacao from the Colombian Amazon
Source: World J Microbiol Biotechnol. 2023 Sep 2;39(11):297. doi: 10.1007/s11274-023-03719-9 (PMC10475004; doi:10.1007/s11274-023-03719-9)
Supplement: Supplementary file 1 — Supplementary file1 (DOCX 102 kb) [file 11274_2023_3719_MOESM1_ESM.docx]

**Supplementary material**

**
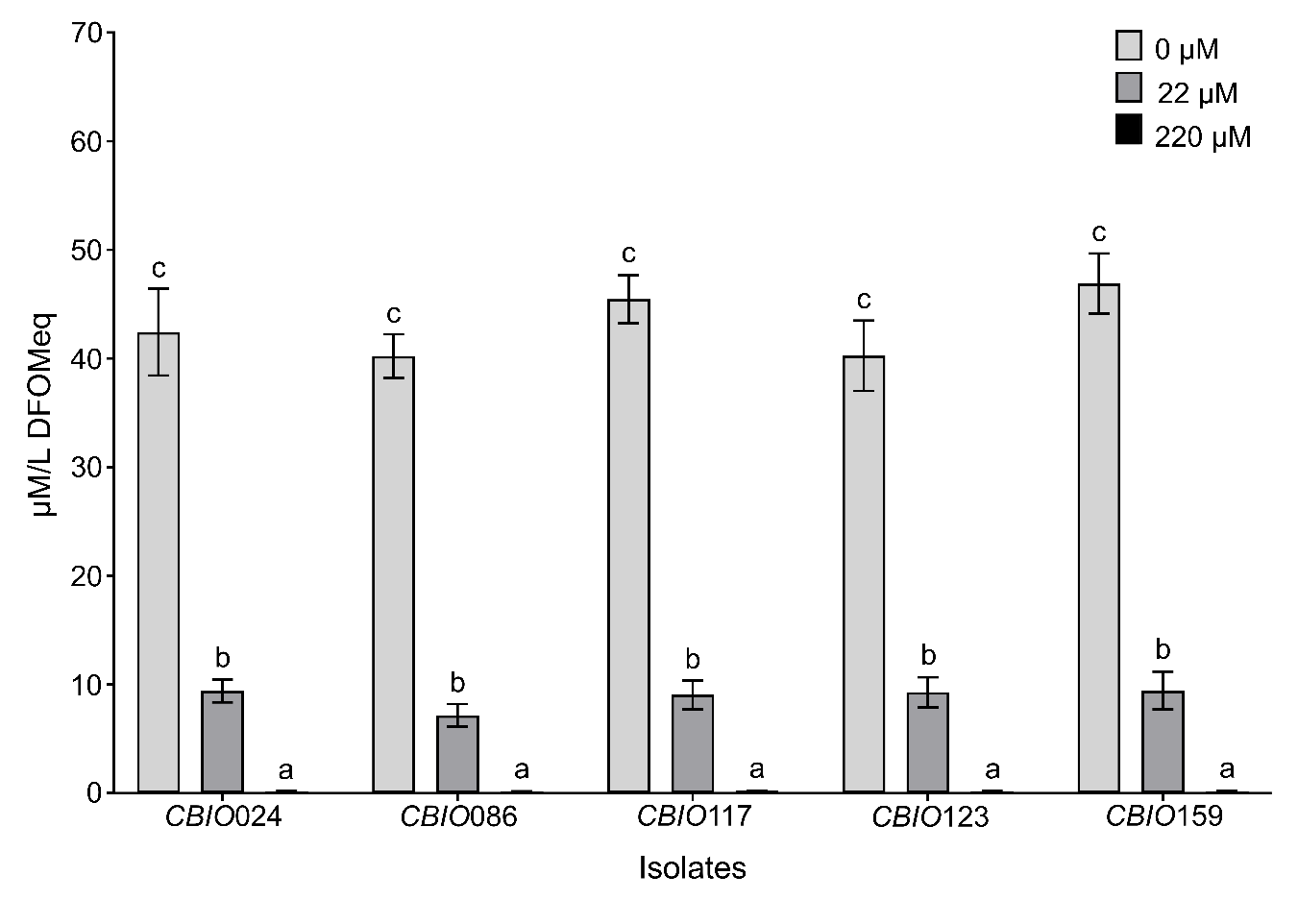
**

**Fig S1** Siderophore production of *A*. *baumannii* isolates in minimal medium without iron and supplemented with FeCl_3._6H*2*O to a final concentration of 22 µM and 220 µM. Values followed by the same letter do not differ significantly (Tukey test, *P*<0.05), values correspond to the mean (± ES) of three biological replicates with n=4.

Table S1. Sampling site coordinates

| Bacterial strain | Latitude | Longitude | Wild species cocoa |
| --- | --- | --- | --- |
| *CBIO*021 | 0,6158 | -74,3653 | *Theobroma subincanum* Mart. |
| *CBIO*024 | 0,1622 | -74,3012 | *Theobroma subincanum* Mart. |
| *CBIO*086 | 0,1622 | -74,3012 | *Theobroma subincanum* Mart. |
| *CBIO*101 | 0,1623 | -74,301 | *Theobroma subincanum* Mart. |
| *CBIO*116 | 0,6475 | -74,5177 | *Theobroma subincanum* Mart. |
| *CBIO*117 | 0,4529 | -75,227 | *Theobroma subincanum* Mart. |
| *CBIO*118 | 0,6075 | -74,3643 | *Theobroma subincanum* Mart. |
| *CBIO*120 | 0,4531 | -75,2278 | *Theobroma subincanum* Mart. |
| *CBIO*121 | -0,0549 | -74,6288 | *Herrania nitida* (Poepp.) R.E. Shult. |
| *CBIO*123 | 0,4531 | -75,2278 | *Theobroma subincanum* Mart. |
| *CBIO*127 | 0,4529 | -75,227 | *Theobroma subincanum* Mart. |
| *CBIO*133 | 0,4779 | -75,1756 | *Theobroma cacao* L. |
| *CBIO*142 | 0,4779 | -75,1756 | *Theobroma cacao* L. |
| *CBIO*149 | -0,0549 | -74,6288 | *Theobroma cacao* L. |
| *CBIO*159 | 0,4773 | -75,1763 | *Theobroma cacao* L. |
